# Acupuncture in Aging-Related Brain Diseases: From Mitochondrial Homeostasis to Clinical Signals

**DOI:** 10.3390/brainsci16101016

**Published:** 2026-09-24

**Authors:** Hang Dong, Wen Wang, Weijian Zeng, Qinglan Zou, Wenhai Lu, Cheng Chen, Junpeng Yao, Ying Li

**Affiliations:** 1 Acupuncture and Tuina School, Chengdu University of Traditional Chinese Medicine, No. 1166 Liutai Avenue, Wenjiang District, Chengdu 611137, China; hangdong@stu.cdutcm.edu.cn (H.D.);; 2 Key Laboratory of Acupuncture for Senile Disease, Ministry of Education, Chengdu University of Traditional Chinese Medicine, No. 1166 Liutai Avenue, Wenjiang District, Chengdu 611137, China

**Keywords:** brain diseases, mitochondrial dysfunction, acupuncture, aging, mitochondrial quality control

## Abstract

**Highlights:**

**What are the main findings?**

- Across animal models of aging-related brain disease, acupuncture is associated with mitochondrial remodeling that spans ultrastructural preservation, biogenesis, dynamics, mitophagy, redox balance, calcium homeostasis, and apoptosis.
- Parallel changes in mitochondrial measures and neural or functional outcomes in experimental animals suggest a potential link between mitochondrial homeostasis and acupuncture-related effects in the aging brain.

**What are the implications of the main findings?**

- Mitochondrial transfer and the mitochondrial unfolded protein response (UPR^mt^) remain insufficiently studied; targeted studies are needed to determine how acupuncture affects these processes in neural tissue and models of aging-related brain disease.
- Clinical studies should pair neurological outcomes with feasible measures of mitochondrial function and energy metabolism to examine whether related biological changes accompany clinical responses.

**Abstract:**

Mitochondria play a key role in maintaining cellular homeostasis through energy metabolism, ROS regulation, calcium handling, and apoptosis control. Mitochondrial dysfunction contributes to neurological vulnerability in the aging brain, where neuronal energy demand, axonal transport, synaptic activity, and neurovascular repair depend on mitochondrial integrity. Animal studies in chronic brain disease and acute brain-injury models have examined acupuncture-related changes in phenotypic outcomes and mitochondrial measures, including behavioral, physiological, and molecular readouts. Their findings span mitochondrial ultrastructure, biogenesis, dynamics, mitophagy, and downstream signaling. Clinical studies have examined acupuncture in Parkinson’s disease, stroke, and Alzheimer’s disease. Animal studies report acupuncture-related changes in mitochondrial structure and indices of quality control alongside improvements in neurological, behavioral, or injury-related outcomes. These mitochondrial changes may influence oxidative stress, calcium regulation, apoptosis, mitochondrial transport, and intercellular mitochondrial support. Clinical studies mainly report symptom and functional outcomes, while a small number of imaging studies also describe changes in cerebral glucose metabolism or neural activity. These preliminary clinical and metabolic signals may inform further investigation into whether mitochondrial processes are associated with clinical responses to acupuncture. Current experimental work also has important limitations: most studies focus on a limited set of mitochondrial quality-control markers, while the mitochondrial unfolded protein response, axonal mitochondrial trafficking, and dynamic mitochondrial transfer are rarely assessed. Future clinical studies should pair neurological outcomes with feasible measures of mitochondrial function and energy metabolism. Tracking these measures over time may help clarify how changes in mitochondrial function and energy metabolism during acupuncture treatment relate to functional recovery.

## 1. Introduction

Mitochondria are central regulators of cellular energy metabolism in eukaryotic cells. Their best-known function is to generate adenosine triphosphate (ATP) through oxidative phosphorylation (OXPHOS), but they also act as sensors of intracellular and extracellular stress. Through this dual role, mitochondria contribute to apoptosis, signal transduction, metabolic adaptation, and other processes that shape cellular homeostasis [1].

Aging is marked by progressive loss of physiological reserve, increased vulnerability to disease, and reduced capacity to maintain homeostasis [2]. Many chronic conditions become more common or more disabling with age, including neurodegenerative and cardio-cerebrovascular disorders [3,4]. As population aging accelerates, neurological degeneration and cerebrovascular injury create a growing clinical and public health burden [5]. Mitochondrial dysfunction is one biological feature that links aging to tissue degeneration. Defects in ATP production, reactive oxygen species (ROS) handling, mitophagy, calcium regulation, and mitochondrial gene expression can all contribute to age-related pathology [6,7]. In the aging brain, where synaptic activity, axonal transport, neuronal survival, and neurovascular repair depend on mitochondrial integrity, declining mitochondrial homeostasis can influence both the progression of chronic brain disease and the response to acute injury.

Acupuncture has been examined as a non-pharmacological intervention in a wide range of clinical conditions, including neurological disorders, chronic pain, gastrointestinal symptoms, and immune-mediated disease, with a relatively low incidence of adverse events in clinical use [8,9,10,11]. In aging-related brain diseases, it is usually evaluated as an adjunctive intervention rather than as a stand-alone disease-modifying treatment. Mechanistic studies have reported acupuncture-associated changes in neuroendocrine-immune networks, inflammation, analgesia, neuroplasticity, and BDNF-related signaling in nervous system diseases [12,13,14]. These system-level effects do not fully explain how peripheral stimulation may influence neuronal metabolism and cellular repair. Mitochondria provide a biologically plausible point of convergence because they integrate energy demand, stress signaling, redox status, and cell-death pathways. Examining how acupuncture-associated mitochondrial responses relate to neural vulnerability and recovery in aging-related brain diseases may clarify the organelle-level processes involved. This review summarizes current evidence on acupuncture-associated mitochondrial changes in aging-related brain diseases and examines their potential clinical significance, with the aim of guiding future mechanistic and clinical research.

## 2. Literature Search and Evidence Selection

A literature search was conducted in PubMed, Web of Science Core Collection, the China National Knowledge Infrastructure (CNKI), the VIP Database, and Wanfang Data from database inception to June 2026. Search terms were adapted for each database and combined acupuncture-related terms (acupuncture, electroacupuncture, manual acupuncture, and acupoint stimulation) with terms for mitochondria and mitochondrial homeostasis (mitochondria, mitochondrial dysfunction, biogenesis, dynamics, mitophagy, oxidative stress, calcium homeostasis, apoptosis, transport, and transfer). Corresponding Chinese terms were used in the Chinese-language databases. Further searches were conducted to identify relevant clinical studies of acupuncture. Mechanistic studies were included when they examined acupuncture or related acupoint stimulation in models of brain aging, neurological disease, or brain injury and reported mitochondrial structure, function, or associated molecular changes. Clinical studies were included when they evaluated acupuncture in patients with neurological disease or brain injury and reported symptom, functional, metabolic, or imaging outcomes. Experimental studies were reviewed to summarize acupuncture-related mitochondrial changes and proposed mechanisms in relevant disease models, and clinical studies were reviewed to describe current evidence and unanswered questions concerning acupuncture for aging-related brain diseases.

## 3. Mitochondrial Ultrastructure and Homeostasis

### 3.1. Mitochondrial Ultrastructure

Mitochondria are abundant in cells with high energy demand and maintain extensive contact with other organelles, including the endoplasmic reticulum (ER) and lipid droplets [15,16,17]. Their double-membrane architecture supports both bioenergetic and signaling functions. The inner mitochondrial membrane (IMM) is the main site of energy metabolism. The outer mitochondrial membrane (OMM), by contrast, provides a permeable interface between mitochondria and the cytosol. The IMM encloses the mitochondrial matrix and folds inward to form cristae. These cristae contain respiratory-chain complexes and ATP synthase, making them essential for OXPHOS [18,19]. The OMM communicates with the cytosol through voltage-dependent anion channels and participates in membrane contact sites with other compartments. These contact sites support fission, fusion, mitochondrial trafficking, and signal relay [20,21].

Mitochondrial compartments also organize molecular functions. The matrix contains circular mitochondrial DNA (mtDNA), which encodes core subunits of respiratory complexes. Proteins translated from mtDNA contribute to Complex I, Complex III, Complex IV, and Complex V, all of which form key parts of the oxidative phosphorylation system [22]. Cristae shape is regulated in part by optic atrophy protein 1 (OPA1), which supports cristae stability, mitochondrial fusion, and inner-membrane remodeling [23]. The OMM contains proteins that control fission, fusion, mitophagy, transport, and apoptosis. These include mitochondrial fission 1 protein (Fis1), mitochondrial fission factor (Mff), MiD49, MiD51, Miro1, mitofusins 1/2 (Mfn1/2), B-cell lymphoma/leukemia-2 protein (BCL-2), BCL-2-associated X protein (BAX), and BCL-2 antagonist/killer (BAK) [21]. Mitochondrial structure determines how mitochondria generate energy, exchange signals, and respond to cellular stress.

### 3.2. Mitochondrial Homeostasis

Mitochondrial homeostasis depends on coordinated control of mitochondrial abundance, morphology, and clearance (Figure 1). Biogenesis increases mitochondrial number and renews mitochondrial components when metabolic demand changes. This process is regulated by both mitochondrial and nuclear genomes and is driven by peroxisome proliferator-activated receptor gamma coactivator 1α (PGC-1α), nuclear respiratory factor 1 (NRF1), nuclear respiratory factor 2 (NRF-2/GABP), and mitochondrial transcription factor A (TFAM). Together, these factors promote mitochondrial gene transcription and improve mitochondrial functional capacity [24,25].

Mitochondrial morphology is maintained through fission and fusion. Fission separates damaged or functionally distinct mitochondrial segments and is mainly driven by dynamin-related protein 1 (Drp1). Receptors and adaptors such as Fis1, Mff, MiD49, and MiD51 recruit Drp1 to the OMM, where GTP hydrolysis promotes membrane constriction [21,27]. Fusion allows mitochondria to exchange contents and complement functional defects. OMM fusion depends on Mfn1/2, whereas IMM fusion is regulated by OPA1 [28]. The balance between these processes determines mitochondrial shape, number, intracellular distribution, and resilience under stress.

Damaged mitochondria are removed through mitophagy. The canonical ubiquitin-dependent pathway is mediated by PTEN-induced kinase 1 (PINK1) and Parkin. When mitochondria lose membrane potential, PINK1 accumulates on the OMM and recruits Parkin, which ubiquitinates OMM proteins such as Mfn1/2. Autophagy adaptors then connect ubiquitinated mitochondria to autophagosomal membranes [29]. Ubiquitin-independent mitophagy is mediated by OMM receptors including BNIP3, BNIP3L, and FUNDC1, especially under stress conditions such as hypoxia [29]. Proteostatic stress can also activate the mitochondrial unfolded protein response (UPRmt), which increases mitochondrial chaperones and proteases through retrograde signaling to the nucleus [30]. Under injury conditions, mitochondria may also be transferred between cells through tunneling nanotubes, extracellular vesicles, gap junction channels, and cell fusion [31].

These quality-control mechanisms support mitochondrial metabolism and signaling. During OXPHOS, electrons from reduced nicotinamide adenine dinucleotide (NADH) and reduced flavin adenine dinucleotide (FADH_2_) pass through the electron transport chain, generating a proton gradient that drives ATP synthesis [32]. Mitochondria also connect glycolysis and lipid metabolism: pyruvate is converted to acetyl-CoA by pyruvate dehydrogenase, and fatty acid β-oxidation occurs in the matrix [33,34]. Beyond bioenergetics, mitochondria sense and transmit signals through ROS, calcium ion (Ca^2+^) flux, mtDNA release, and metabolites such as succinate. These signals influence apoptosis, inflammation, epigenetic regulation, and quality-control pathways such as PINK1/Parkin mitophagy [35].

## 4. Mitochondria in Aging-Related Brain Diseases

Mitochondrial dysfunction is a recurrent feature of aging, but its consequences are not the same in every tissue. With age, mitochondrial oxidative phosphorylation (OXPHOS) capacity declines most clearly in tissues with high energy demand, including the brain, heart, and skeletal muscle [36,37]. This decline often appears alongside mtDNA mutations, respiratory-chain dysfunction, excess ROS generation, and impaired quality control [38]. In the aging nervous system, these changes carry particular weight because neuronal activity depends on sustained ATP production, mitochondrial positioning along axons and synapses, Ca^2+^ buffering, and timely removal of damaged organelles. Mitochondrial dysfunction is now recognized as a central contributor to aging-related brain diseases, especially neurodegenerative disease, where impaired bioenergetics, oxidative injury, calcium stress, and defective mitophagy converge on neuronal vulnerability [39,40]. These mitochondrial changes can become self-amplifying. Damaged mitochondria are major sources of ROS, and when ROS production exceeds antioxidant capacity, DNA, proteins, lipids, and organelles are injured [41]. Excess ROS can also promote the senescence-associated secretory phenotype, worsening cellular senescence, proteostatic stress, and epigenetic instability [42]. In this setting, mitochondrial damage is both a marker of aging and a driver of further tissue decline: ROS damages mtDNA and weakens respiratory-chain function, while inflammatory mediators further reduce bioenergetic capacity [38].

The nervous system is especially vulnerable to this pattern because neuronal function depends heavily on mitochondrial energy production and quality control. Mitophagy does not, however, decline uniformly with age. Basal mitophagy remained active across multiple brain regions in healthy aging mice, indicating that age-related changes depend on brain region, cell type, and pathological context [43]. In Parkinson’s disease (PD), dopaminergic neuron degeneration and α-synuclein aggregation are central pathological features [44]. Aggregated α-synuclein damages mitochondria, disrupts OXPHOS and ATP production, and promotes retention of damaged mitochondria by binding to the OMM protein Miro [45,46].

Alzheimer’s disease (AD) shows a related but not identical mitochondrial profile. Mitochondrial energy insufficiency and excess ROS accompany Aβ and tau pathology and contribute to oxidative injury [47]. Familial Alzheimer’s disease has also been associated with altered ER-mitochondria coupling, reduced Ca^2+^-signaling molecules, and lower levels of mitochondrial motility proteins such as MIRO1 and KIF5C [48]. Together, these findings place mitochondrial dysfunction near several core processes of neurodegeneration rather than at a single downstream endpoint.

Stroke illustrates how acute injury can expose the same mitochondrial vulnerabilities. Ischemia suppresses OXPHOS and rapidly lowers ATP levels. Ion-pump failure then promotes Ca^2+^ overload, while reperfusion turns mitochondria into a major source of ROS [49]. The combination of ROS and Ca^2+^ overload collapses the mitochondrial membrane potential and activates apoptotic pathways. Stroke also damages the mitochondrial genome and changes mitochondrial content at the tissue level. In animal models, mtDNA levels after ischemia–reperfusion influence recovery [50]. Clinically, circulating cell-free DNA is elevated in acute ischemic stroke and has been associated with innate immune activation [51]. But that study did not specifically quantify the mitochondrial fraction, and thus it does not establish circulating mtDNA as a stroke biomarker.

## 5. Acupuncture Targeting Mitochondrial Homeostasis

### 5.1. Mitochondrial Ultrastructure

Mitochondrial ultrastructure is closely tied to mitochondrial function. Aging-related stress can produce swelling, cristae fragmentation, membrane disruption, and distorted morphology. These structural changes impair bioenergetic function and can amplify downstream injury. Although similar abnormalities appear across diseases, their pattern and severity depend on the model, tissue type, and stage of injury.

In experimental models of acute ischemic brain injury, acupuncture has been reported to preserve mitochondrial ultrastructure. In rats with cerebral ischemia–reperfusion injury, electroacupuncture at Baihui (GV20) and Dazhui (GV14) reduced mitochondrial vacuolization and cristae swelling, with better preservation of the double-membrane structure [52]. Electroacupuncture preconditioning at GV20 also attenuated mitochondrial swelling in the ischemic penumbra. Quantitative analysis showed a higher mitochondrial aspect ratio and a lower proportion of vacuolated mitochondria than in untreated ischemia–reperfusion rats [53]. In a rat model of acute ischemic stroke, delayed rtPA thrombolysis combined with Xingnao Kaiqiao acupuncture at bilateral PC6 and Shuigou (GV26) reduced mitochondrial shrinkage, cristae loss, and OMM rupture compared with delayed rtPA thrombolysis alone [54].

These structural effects are not limited to acute injury. In chronic disease models, mitochondrial damage can include vacuolization, blurred membranes, and progressive distortion. Electroacupuncture preserved mitochondrial ultrastructure in striatal neurons of MPTP-induced PD mice, with fewer damaged mitochondria and better-defined cristae [55]. In Aβ25–35-induced AD mice, electroacupuncture alleviated hippocampal mitochondrial damage characterized by swollen or disrupted cristae and ruptured membranes, alongside improvements in learning and memory [56]. Electroacupuncture also reduced mitochondrial swelling and preserved outer membrane integrity and cristae organization in hippocampal neurons of APP/PS1 mice, accompanied by a higher mitochondrial membrane potential and improved learning and memory [57]. These observations support an association between acupuncture and mitochondrial structural preservation in the examined models, but most studies still rely on qualitative electron-microscopy assessment rather than standardized morphometric measures.

### 5.2. Mitochondrial Biogenesis

Mitochondrial biogenesis renews mitochondrial components and increases mitochondrial abundance when cellular energy demand changes. PGC-1α is a central regulator of this process and also contributes to mitochondrial quality control. During aging, impaired PGC-1α signaling is associated with brain aging, neurodegenerative disease, metabolic dysregulation, and chronic pain [58,59,60]. The same pathway also regulates energy metabolism, antioxidant responses, and inflammation [61]. AMPK provides a major route through which energy stress is translated into mitochondrial renewal. As an energy-sensing kinase, AMPK coordinates several steps of mitochondrial quality control. During energy stress, it induces PGC-1α, which acts with NRF1, NRF-2/GABP, and TFAM to activate mitochondrial gene transcription [62]. Experimental studies have reported activation of this axis and changes in mitochondrial-biogenesis markers after acupuncture in aging-related brain disease and brain-injury models (Table 1).

Acupuncture-associated changes in mitochondrial biogenesis have also been reported outside the canonical AMPK pathway. Cannabinoid receptor-1 (CB-1R), a G-protein-coupled receptor on the plasma membrane, regulates neuronal signaling and synaptic plasticity in the brain. Evidence also links cannabidiol and CB-1R signaling to mitochondrial biogenesis in neuronal and non-neuronal tissues [70,71]. In a mouse model, electroacupuncture at Baihui (GV20) upregulated CB-1R, increased the expression of the biogenesis-related proteins NRF1 and TFAM, and reduced the model-associated increase in cytochrome c oxidase subunit-IV (COX-IV) expression. These changes were associated with reduced post-stroke depressive-like behavior and improved cognition. CB-1R antagonism with AM251 or CB-1R knockdown attenuated these behavioral improvements and the increases in NRF1 and TFAM expression [72]. Another study reported that electroacupuncture preconditioning at GV20 increased PGC-1α-related signaling, mitochondrial-biogenesis markers, mitochondrial membrane potential, and ischemic tolerance. CB-1R antagonism or PGC-1α knockdown attenuated the increases in NRF1 and TFAM expression and the improvement in mitochondrial membrane potential [73].

Beyond AMPK and CB-1R, NAD+-dependent SIRT1 provides another regulatory route to PGC-1α and mitochondrial adaptation during aging. NAD+ availability supports mitochondrial redox reactions and energy metabolism while sustaining sirtuin activity; its age-related decline may therefore compromise SIRT1-dependent control of mitochondrial homeostasis [74]. Mechanistic work in a premature-aging model further showed that SIRT1-mediated deacetylation of PGC-1α depends on coordinated interactions with TFIIH at metabolic gene promoters, and that disruption of this partnership impairs the corresponding transcriptional response [75]. In senescence-accelerated mice, acupuncture at GV20, Xuehai (SP10), Shenshu (BL23), and Geshu (BL17) upregulated hippocampal SIRT1–PGC-1α signaling, increased mitochondrial membrane potential, and reduced neuronal injury [76]. These findings place SIRT1–PGC-1α signaling alongside AMPK and CB-1R as another candidate route through which acupuncture may support mitochondrial function during brain aging.

### 5.3. Mitochondrial Dynamics

Mitochondrial dynamics are governed by fission and fusion. Disruption of this balance can alter mitochondrial morphology, impair quality control, and influence apoptosis. The apoptotic link is mediated largely through mitochondrial outer membrane permeabilization. Fission machinery, including Drp1, can shape apoptotic efficiency by affecting post-MOMP remodeling and cytochrome c release. By contrast, BCL-2 family-mediated MOMP is the central commitment step for cytochrome c-dependent caspase activation [77]. OPA1-dependent cristae remodeling adds another point of control. OPA1 processing generates membrane-anchored long forms and soluble short forms, and both contribute to cristae organization, although their effects on inner-membrane fusion and cristae architecture are context dependent [23]. When apoptotic stimuli disrupt OPA1 oligomerization or proteolytic balance, crista junctions can widen and cytochrome c release becomes more complete, promoting apoptosis [78]. Across the available studies, improvements after acupuncture were accompanied by coordinated changes in fission- and fusion-related measures rather than a uniform change in either process alone. Reduced mitochondrial fragmentation and changes in fusion-related proteins are consistent with recovery toward a more balanced mitochondrial network. In ischemic and hemorrhagic stroke models, this pattern was reflected by reduced expression of Drp1 or Fis1 and, in studies assessing both processes, increased expression of Mfn1/2 and changes in OPA1 expression or its short-to-long-form ratio (Table 1). These molecular changes were accompanied by improved mitochondrial morphology and function, reduced neuronal injury or apoptosis, and better neurological or cognitive outcomes. In rats with cerebral ischemia–reperfusion, Chen et al. found that electroacupuncture increased hippocampal SIRT1, PGC-1α, and OPA1 while suppressing Drp1. The intervention reduced neuronal and mitochondrial damage, decreased mitochondrial fragmentation, and improved learning and memory deficits [79]. Taken together, these findings suggest that the reported mitochondrial recovery after acupuncture is accompanied by coordinated remodeling of fission and fusion, with changes in PGC-1α-related signaling in selected models.

### 5.4. Mitophagy-Related Responses

Mitophagy removes damaged mitochondria and prevents the accumulation of ROS, inflammatory signals, and cell-death triggers. It occurs mainly through ubiquitin-dependent and ubiquitin-independent pathways. The ubiquitin-dependent pathway centers on PINK1/Parkin signaling. PINK1 and Parkin interact with p62, Optineurin (OPTN), and microtubule-associated protein 1 light chain 3 (LC3) to target damaged mitochondria for autophagic degradation [80].

After mitochondrial depolarization, PINK1 cleavage by inner-membrane proteases stops, allowing PINK1 to accumulate on the OMM. PINK1 then autophosphorylates, phosphorylates ubiquitin chains, recruits and activates Parkin, and promotes ubiquitination of OMM proteins such as Mfn1/2 [81]. Autophagy adaptors recognize these chains and link damaged mitochondria to the autophagosomal membrane. This interaction supports mitophagosome formation and subsequent fusion with lysosomes for degradation. p62 and LC3 are commonly measured in autophagy and mitophagy studies [82]. Acupuncture-related changes in mitophagy vary across experimental models. Most neurological studies have focused on PINK1/Parkin-related signaling (Table 1). In a rat model of aging induced by D-galactose and chronic unpredictable stress, acupoint catgut embedding at GV20, ST36, LI4, and LR3 increased hippocampal PINK1 and LC3 and reduced p62, whereas Parkin expression did not change significantly. Improved mitochondrial morphology and learning performance were accompanied by increased autophagic activity assessed using LC3 dual-fluorescence imaging. The partial attenuation of the fluorescence response and behavioral improvements by 3-methyladenine (3-MA) supported the involvement of autophagy [83]. In rats with acute embolic stroke receiving delayed rt-PA, acupuncture further increased the already elevated PINK1, Parkin, and LC3 levels and reduced p62 in the ischemic penumbra. Electron microscopy showed more mitophagic structures and better-preserved mitochondrial ultrastructure. Mdivi-1 attenuated the changes in mitophagy-related markers and mitochondrial morphology [67]. In a 6-OHDA-induced PD mouse model, electroacupuncture initiated 30 days after lesion induction reduced the elevated PINK1, Parkin, and Miro1 levels and increased KIF5A and p62 in the prefrontal cortex. These changes were accompanied by fewer autophagosomes, improved mitochondrial morphology, and better cognitive performance [69]. Acupuncture-related improvements in mitochondrial morphology were accompanied by divergent changes in mitophagy-related markers [67,69]. Findings from autophagy assays and inhibitor experiments provide additional support for autophagy involvement [67,83], while their relationship to mitochondrial clearance awaits further clarification. The direction of individual markers therefore does not by itself establish whether mitochondrial clearance was increased, suppressed, or normalized.

Ubiquitin-independent mitophagy is initiated by OMM receptors that bind directly to LC3, bypassing the need for ubiquitination. BNIP3 and FUNDC1 are the best-characterized receptors in this pathway. During hypoxia or stress, BNIP3 forms dimers on the OMM and binds LC3. This promotes Drp1 activation and mitochondrial fission while inhibiting OPA1-mediated fusion [84]. Hypoxia also induces FUNDC1 dephosphorylation, which strengthens its interaction with LC3B. FUNDC1 accumulates at ER-mitochondria contact sites and facilitates Drp1-mediated fission [85].

Recent studies in ischemic brain injury models have begun to characterize acupuncture-related changes in these ubiquitin-independent receptor pathways. In rats with cerebral ischemia, electroacupuncture at GV20 and PC6 increased HIF-1α, BNIP3, Beclin-1, and LC3-II expression and autophagosome formation. These changes were accompanied by less mitochondrial damage, smaller infarct areas, and lower neurological deficit scores. The similar response observed in DMOG-treated rats was consistent with the involvement of HIF-1α/BNIP3-related signaling [86]. FUNDC1 has been examined in studies that also included pharmacological intervention. In rats with cerebral ischemia–reperfusion injury, electroacupuncture pretreatment at GV20 and GV26 reduced injury-associated increases in FUNDC1, p-ULK1, LC3-II, and autolysosome numbers while preserving mitochondrial structure and membrane potential. Rapamycin attenuated these effects, implicating mTORC1/ULK1/FUNDC1-related signaling [87]. In a post-stroke treatment model, electroacupuncture initiated 24 h after reperfusion increased PGAM5 and FUNDC1 expression, LC3–TOMM20 colocalization, and autophagic structures in the ischemic penumbra. These changes were accompanied by lower ROS levels and cytosolic cytochrome c, reduced neuronal apoptosis, and improved neurological function. 3-MA attenuated several of these effects [88]. Together, these studies extend acupuncture-related mitophagy research beyond ubiquitin-dependent PINK1/Parkin signaling to include BNIP3- and FUNDC1-related pathways of ubiquitin-independent mitochondrial clearance.

### 5.5. Mitochondrial Redox, Calcium, and Apoptotic Signaling

Acupuncture studies in neural injury models have reported changes in mitochondrial redox regulation, Ca^2+^ handling, and intrinsic apoptotic signaling. These processes are closely interconnected. Excess ROS damages respiratory proteins, mtDNA, lipids, and mitochondrial membranes; Ca^2+^ overload destabilizes membrane potential and promotes cytochrome c release; and sustained mitochondrial stress engages intrinsic apoptotic signaling. Redox stress also weakens respiratory and antioxidant capacity, while Ca^2+^-linked signaling has been linked to organelle stability and mitochondrial transfer. Across models of ischemic stroke, vascular dementia, PD, traumatic brain injury (TBI), and postoperative cognitive dysfunction (POCD), the reported changes span antioxidant capacity, respiratory-chain activity, mitochondrial transfer, Ca^2+^-linked transport, membrane potential, mitochondrial dynamics, and caspase activation (Figure 2).

Mitochondria are a major intracellular source of ROS, and their redox state is shaped by oxidant production and antioxidant defenses. MDA is commonly used as an index of lipid peroxidation, whereas SOD, glutathione peroxidase, and catalase reflect complementary antioxidant processes [22,89,90]. Acupuncture studies in dementia and ischemic stroke models have reported changes in mitochondrial respiration alongside these redox indices. In multi-infarct dementia rats, acupuncture increased total SOD, Cu/Zn-SOD, and Mn-SOD activities, lowered mitochondrial MDA and superoxide levels, increased the GSH/GSSG ratio, and improved mitochondrial respiratory control, the P/O ratio, respiratory enzyme activities, cerebral blood flow, and cognitive performance [91]. In vascular dementia rats, acupuncture at GV20 and bilateral ST36 increased hippocampal mitochondrial complex I, II, and IV activities, cytochrome c oxidase IV expression, respiratory control, and membrane potential while reducing ROS generation and improving learning and memory [92]. In focal ischemic stroke, laser acupuncture at GV20 reduced infarct volume and cortical mitochondrial MDA levels while increasing mitochondrial SOD, catalase, and glutathione peroxidase activities [93]. Baihui laser acupuncture also increased hippocampal mitochondrial SOD and hippocampal glutathione peroxidase activities, accompanied by higher neuronal density in the CA1 and CA3 regions and better cognitive and motor performance [94]. In MCAO rats, acupuncture increased hippocampal PGC-1α, NRF1, and TFAM expression, oxidative phosphorylation complexes I, IV, and V, and ATP levels while preserving mitochondrial membrane potential. These mitochondrial and bioenergetic changes occurred alongside an increased GSH/GSSG ratio and reduced MDA levels, while SOD activity showed a nonsignificant upward trend [95]. Respiratory control and P/O measurements in the dementia models provide more direct evidence of mitochondrial functional improvement than antioxidant indices alone. These findings suggest that reduced oxidative stress after acupuncture is accompanied by improved coupling of mitochondrial respiration to ATP production.

Other studies have examined oxidative injury together with inflammatory, ferroptotic, and autophagy-related signaling. In vascular dementia rats, electroacupuncture at GV20, GV14, and BL23 reduced hippocampal ROS levels, NLRP3 and Beclin-1 expression, and the LC3-II/LC3-I ratio while improving learning and memory and reducing CA1 neuronal ultrastructural damage [96]. In MPTP-treated mice, acupuncture at GB34 reduced lipid peroxidation and mitochondrial abnormalities and preserved TH-positive dopaminergic neurons [97]. In another PD model, electroacupuncture reduced oxidative and inflammatory markers, improved mitochondrial membrane potential, and altered intracellular Ca^2+^ levels together with TRPC1 and SIRT1/AMPK signaling. TRPC1 knockdown suppressed SIRT1/AMPK signaling, increased neuronal apoptosis, and attenuated the improvement in mitochondrial membrane potential. SIRT1 or AMPK inhibition also weakened the membrane-potential response to electroacupuncture [98]. In ischemic stroke, electroacupuncture reduced Fe^2+^, ROS, and MDA accumulation, increased SOD, GPX4, SLC7A11, and FTH1, and preserved mitochondrial ultrastructure. The Nrf2 inhibitor Brusatol attenuated the reductions in ROS and MDA and the preservation of mitochondrial ultrastructure, as well as the neuroprotective effect, supporting the involvement of Nrf2 signaling in the observed anti-ferroptotic response [99]. In aged rats with postoperative cognitive dysfunction, electroacupuncture pretreatment reduced hippocampal ROS and serum MDA, increased serum SOD and catalase levels, and improved cognitive performance and neuronal and mitochondrial ultrastructure. These changes occurred alongside increased SIRT1 and Beclin-1 and reduced FOXO1 and p62 expression [100].

Ca^2+^ signaling links mitochondrial metabolism with injury signaling. Physiological mitochondrial Ca^2+^ uptake supports tricarboxylic acid cycle activity and ATP synthesis, whereas Ca^2+^ overload promotes permeability-transition pore opening, membrane-potential collapse, cytochrome c release, and apoptosis. Ca^2+^ has a dual role: controlled Ca^2+^ flux supports mitochondrial activity and intercellular mitochondrial supply, whereas excessive Ca^2+^ flux lowers the threshold for mitochondrial failure and cell death. In an acute ischemic stroke model, electroacupuncture promoted astrocyte-to-neuron mitochondrial transfer, increased functional mitochondria within neurons, and reduced neuronal apoptosis. These effects were accompanied by astrocytic CD38 upregulation and changes in cADPR/Ca^2+^ signaling and TNT-associated proteins, including F-actin, Miro1, TRAK1, and KIF5b, suggesting their potential involvement in mitochondrial transfer [101]. This places Ca^2+^ at the interface between glial support and neuronal mitochondrial replenishment. Channel and purinergic signaling form another Ca^2+^-linked injury site. In a TBI model, electroacupuncture reduced PANX1 expression, Ca^2+^ levels, cytochrome c protein levels, caspase-3 activation, oxidative stress, and apoptosis, while increasing ATP levels and mitochondrial respiratory-chain activity. In a separate group, the PANX1 inhibitor probenecid produced similar ATP and Ca^2+^ changes and neurological improvements, supporting a possible role for PANX1 in the effects of electroacupuncture [102].

Mitochondrial apoptosis is closely linked to neuronal loss, and acupuncture studies have examined this process across several neural injury models. In focal cerebral ischemia–reperfusion rats, electroacupuncture at GV26 and GV20 increased cortical mitochondrial membrane potential and reduced neuronal apoptosis [103]. In MCAO rats, 2-Hz electroacupuncture reduced infarct area and TUNEL-positive cells, decreased DR5 expression and caspase-3 and caspase-8 activity, and increased Bcl-2, Bcl-xL, cIAP-1, and cIAP-2 expression [104]. Related changes in Cyt-C and caspase signaling were observed in TBI and POCD models. Electroacupuncture reduced Cyt-C and caspase-9 expression together with neuronal apoptosis after TBI [105]. In aged rats exposed to anesthesia and surgery, electroacupuncture pretreatment improved cognition and mitochondrial ultrastructure while reducing cytosolic Ca^2+^, mPTP opening, ROS, Cyt-C, cleaved caspase-9 and caspase-3, the Bax/Bcl-2 ratio, and hippocampal neuronal apoptosis [106]. Reduced apoptosis was also accompanied by changes in mitochondrial fission and protein translocation. Electroacupuncture preconditioning at GV20 decreased total and mitochondrial Drp1 expression, preserved mitochondrial morphology, and reduced apoptosis in the ischemic penumbra [53]. Post-ischemic electroacupuncture at GV20 and GV24 improved neurological function while reducing infarct volume, cofilin translocation to mitochondria, caspase-3 cleavage, and neuronal apoptosis [107]. Across these experimental studies, acupuncture-related reductions in neuronal apoptosis were reported alongside changes in mitochondrial integrity, apoptotic signaling, mitochondrial fission, and protein translocation.

## 6. Neural Mitochondrial Control Points in Acupuncture Studies

Experimental studies associate acupuncture with preservation of mitochondrial structure and changes in biogenesis, dynamics, mitophagy, and stress signaling (Figure 3). The functional significance of these changes depends on how the removal of damaged organelles is balanced with the preservation of local energy supply. Different patterns of injury and metabolic demand may therefore produce distinct molecular responses despite improvements in neuronal function.

### 6.1. Parkinson’s Disease

Improved mitochondrial morphology in PD models has accompanied both increases and decreases in PINK1–Parkin expression. EA increased previously reduced PINK1 and Parkin expression in the substantia nigra of MPTP-treated mice, alongside improved motor performance [108]. In 6-OHDA-lesioned mice, improvements in prefrontal mitochondrial morphology and cognition instead accompanied decreases in elevated PINK1 and Parkin expression [69]. Treatment began during MPTP administration in the former study and 30 days after lesion induction in the latter. Brain region, toxin exposure, and treatment timing varied together, so the opposing responses cannot be attributed to disease stage alone. PINK1–Parkin signaling responds to mitochondrial damage [109]. Where damage recognition or recruitment for clearance is insufficient, increased signaling may favor removal. If treatment reduces mitochondrial injury, lower signaling may reflect reduced clearance demand. High marker abundance can also persist when downstream degradation is incomplete. Structural recovery could thus accompany different marker profiles, depending on the balance between damage burden and clearance capacity. Neither PD study measured clearance flux, leaving the relative contributions of these processes unresolved.

Clearance demand also depends on the rate at which mitochondria are damaged. In rotenone-treated rats, EA altered fecal metabolites and serum lipids. Untreated model animals had higher serum plasmenylethanolamine but lower nigral levels than controls, whereas EA increased nigral plasmenylethanolamine and reduced local lipid peroxidation [110]. The handling of damaged mitochondria also involves their transport. Removal of Miro can arrest the movement of damaged mitochondria before degradation, allowing transport and clearance to be coordinated [111]. The same prefrontal study that reported lower PINK1 and Parkin expression also found reduced Miro1 and increased KIF5A, providing expression-level evidence of a response in transport-related machinery [69]. Changes in mitochondrial organization have also been observed at ER contact sites. In the substantia nigra of MPTP-treated mice, EA improved ER–mitochondria contacts, with *Fn1* identified as a transcriptomic candidate associated with the response [112]. Limiting oxidative injury may reduce the demand for mitochondrial clearance and help surviving neurons retain a functional mitochondrial pool [110]. How effectively these organelles support neuronal activity also depends on their positioning relative to local energy requirements [111]. The changes in prefrontal transport-related proteins and improvement in nigral ER–mitochondria contacts suggest that EA may influence mitochondrial organization within affected neurons, alongside reducing the burden of damage [69,112]. Maintaining a locally available supply of functional mitochondria could help surviving neurons sustain activity, contributing to the motor and cognitive improvements reported in these PD models.

### 6.2. Ischemic Brain Injury

In ischemic brain injury, treatment timing changes the biological conditions under which acupuncture acts. Pretreatment examines resistance to a subsequent insult, whereas postischemic intervention acts on established damage. In rats, EA pretreatment reduced FUNDC1-related autophagy markers alongside preservation of mitochondrial structure and membrane potential [87]. In a separate study, EA initiated 24 h after reperfusion increased PGAM5/FUNDC1-related markers and reduced neuronal apoptosis [88]. These different directions may reflect reduced clearance demand when pretreatment limits the ensuing injury, but greater recruitment for clearance when damaged organelles are already present. Metabolic changes before ischemia offer a possible basis for limiting the subsequent injury burden. Brain metabolomics showed that EA pretreatment altered metabolism before ischemia and partially reversed subsequent disturbances involving glycolysis, the TCA cycle, and purine metabolism [113]. The glycolytic component was examined more directly in a separate study of lactate production and protein lactylation. EA pretreatment reduced lactate accumulation, protein lysine lactylation, and brain injury after ischemia. No additional benefit was observed when EA was combined with glycolysis inhibition using 2-deoxyglucose [114]. This non-additive response is consistent with overlapping metabolic effects and supports reduced lactate production and lactylation as candidate components of the pretreatment response.

Lactate production may also affect the ability of astrocytes to supply mitochondria to injured neurons. In a non-acupuncture stroke study, astrocytic LRP1 promoted mitochondrial transfer while reducing lactate production and ARF1 lactylation [115]. Acupuncture experiments have separately examined whether astrocytes participate in this form of support. Guo et al. investigated both pretreatment and postischemic EA in rats and reported fluorescence-labeling and coculture observations consistent with astrocyte-to-neuron mitochondrial transfer. The mechanistic coculture experiments used astrocytes obtained from EA-pretreated animals [101]. In a separate pretreatment study, astrocytic Miro1 knockdown attenuated the EA-associated reduction in infarct volume and increases in neuronal survival, ATP content, and TOM40 expression. Improvements in these outcomes were also reported in the Miro1-overexpression group [116]. These cell-specific perturbations support astrocytic Miro1 involvement in neuroprotection, with neuronal ATP and TOM40 serving as readouts of energy status and mitochondrial protein abundance. The astrocytic result concerns support for neighboring neurons, whereas the PD study measured Miro1 abundance within affected prefrontal tissue. Neuronal energy recovery after ischemia may depend on both preservation of neuronal mitochondria and the metabolic capacity of supporting astrocytes. Lower astrocytic lactate production may favor mitochondrial transfer when ARF1 lactylation restricts this process, while lactate delivery to neurons may help sustain their energy supply [115,117]. EA pretreatment may increase ischemic tolerance partly by preserving the capacity of astrocytes to support injured neurons [101,116]. Such support could help sustain neuronal energy supply during the period when ischemia has compromised the neurons’ own mitochondria.

### 6.3. Alzheimer’s Disease

In AD models, hippocampal mitochondrial preservation has accompanied improvements in learning and memory. In an acute Aβ25–35 injection model, these behavioral improvements occurred alongside preservation of mitochondrial ultrastructure and changes in dynamics- and autophagy-related markers [56]. The contribution of an implicated regulatory pathway was tested in APP/PS1 mice by combining measurements of mitochondrial morphology, membrane potential, and energy metabolism with neuronal AMPKα1 conditional knockout. Deletion of AMPKα1 aggravated the underlying pathology and attenuated the mitochondrial and cognitive responses to EA, although some treatment effects persisted [57]. The attenuation of both responses provides functional support for AMPKα1 involvement in hippocampal recovery, beyond the parallel marker and behavioral changes observed in the acute injury model.

Even with better-preserved mitochondria, impaired glucose uptake may continue to restrict hippocampal energy supply. In 5×FAD mice, EA increased hippocampal FDG uptake and neuronal GLUT3 membrane localization, accompanied by higher ATP content and citrate synthase activity. Hippocampal IGF1R knockdown attenuated the metabolic and cognitive responses [118]. No significant changes were detected in the other measured TCA-cycle enzyme activities. In the same study, hippocampal administration of 2-deoxyglucose attenuated the EA-associated improvements in learning and memory [118]. The AMPKα1 and IGF1R findings suggest that mitochondrial preservation and improved neuronal glucose uptake contribute to the cognitive response to EA in the respective AD models [57,118]. By improving glucose utilization, EA may also relieve a metabolic constraint on the function of surviving hippocampal neurons. This could increase the contribution of preserved mitochondria to the energy supply needed for learning and memory. The mitochondrial studies in these disease models have predominantly used EA. The responses observed under a particular protocol may reflect the stimulus delivered as well as the pathological state of the tissue [119].

## 7. Acupuncture Protocols and Specificity

In studies of aging-related brain diseases, acupuncture is delivered through a wide range of treatment protocols. Variation in stimulus modality, acupoint prescription, stimulation parameters, and treatment schedule is one source of the differences in reported treatment effects [119]. The approaches represented in this literature include manual acupuncture (MA), electroacupuncture (EA), scalp acupuncture, laser acupuncture, acupoint catgut embedding, and thermal techniques such as warm-needle acupuncture, fire acupuncture, and moxibustion. These terms describe different aspects of treatment: MA and EA distinguish the mode of needle stimulation, whereas scalp acupuncture specifies the treatment location and may use either method. Moxibustion delivers a thermal stimulus without necessarily involving needle insertion. Table 2 summarizes the principal stimulus and timing characteristics of these interventions.

Stimulus modality and dose are often intertwined. Manual manipulation is usually brief or intermittent, whereas electrical stimulation may continue throughout a session; equal needle retention times therefore need not represent equal periods of active stimulation [119]. Treatment exposure also depends on manipulation or electrical parameters, repeated stimulation within a session, the interval between sessions, and the overall course [120]. Across trials included in a systematic review of acupuncture for Alzheimer’s disease, treatment courses ranged from 20 days to 24 weeks [121]. Differences in treatment effects have been reported even within the same modality. In experimental ischemic stroke, a preclinical meta-analysis yielded different subgroup estimates of EA effects on infarction, apoptosis, and inflammation according to waveform and intervention timing [122]. Clinical comparisons also depend on the outcome assessed. A network meta-analysis in Parkinson’s disease reported outcome-dependent rankings among acupuncture-related regimens, many of which included medication; certainty was low to moderate for most comparisons [123]. For global cognitive outcomes in Alzheimer’s disease and mild cognitive impairment, moderator analyses did not detect a difference between MA and EA [124]. Stimulus modality and dose may therefore affect treatment efficacy and the neural or molecular responses involved in ways that depend on the disease context.

Acupoint selection determines where peripheral stimulation is applied. Among the mitochondrial studies summarized in Table 1, GV20 was used most often, while GV26, ST36, and PC6 each appeared in more than one protocol. The frequent selection of GV20 and ST36 is corroborated by a systematic review of clinical trials in Alzheimer’s disease, in which these were the two most commonly used points; SP6, KI3, BL23, EX-HN1, ST40, LR3, and PC6 were also frequently included [121]. Neuroimaging studies have reported both shared and differing cerebral responses across acupoints, with substantial variation in stimulation methods and study design [125]. In a small fMRI study involving patients with Alzheimer’s disease or mild cognitive impairment, separate within-group comparisons with baseline showed partly distinct patterns of brain activation and deactivation during stimulation at Siguan (bilateral LI4 and LR3) or adjacent sham points [126]. In ischemic-stroke models, study-level subgroup estimates also varied among scalp, body, and combined acupoint prescriptions, offering a preliminary clue to site-dependent neuroprotective and inflammatory responses [122]. Together, these differences may help explain the varying degrees of symptom improvement and functional recovery reported in studies of acupuncture for aging-related brain diseases.

**Table 2:** Stimulus and timing characteristics of acupuncture and related interventions discussed in aging-related brain disease research.

Intervention	Stimulus and Distinguishing Parameters	Timing and Repetition
Manual acupuncture [119,120]	Needle insertion and manual manipulation; insertion depth, manipulation method and intensity, intended response	Needle retention time; duration and frequency of manipulation during retention
Electroacupuncture [119,121]	Electrical stimulation through inserted needles; electrode pairing, waveform, frequency, pulse width, current intensity	Electrical-stimulation time and needle retention time. Example from an AD trial: 30-min retention, three sessions weekly for 12 weeks
Scalp acupuncture [121,122]	Needling of scalp points, lines, or zones; anatomical location and manual or electrical stimulation	Retention time and duration of manual manipulation or electrical stimulation
Laser acupuncture [127]	Nonthermal laser irradiation; wavelength, power, beam area, energy delivered	Exposure time per point; repeated or simultaneous irradiation
Acupoint catgut embedding [83]	Implantation of absorbable material at acupoints; material, amount, and implantation depth	Stimulation extends between visits; timing is characterized by the interval between embedding procedures
Warm-needle acupuncture [123]	Heat applied to an inserted needle; heating method and amount of moxa when used	Needle retention, heating duration, and number of heating applications
Fire acupuncture [123]	Brief insertion of a heated needle; needle characteristics and application sites	Brief individual applications; number of applications per session
Moxibustion [123]	Thermal stimulation from burning moxa; direct or indirect application and distance from the skin	Exposure per point and number of applications

Note: Each protocol is further characterized by its acupoint prescription, laterality, session frequency, total number of sessions, and overall treatment course. Scalp acupuncture may be delivered manually or electrically. AD, Alzheimer’s disease.

## 8. Clinical Signals in Aging-Related Brain Diseases

The neurological and behavioral improvements reported alongside cellular changes in experimental models provide a rationale for examining acupuncture’s potential benefits in aging-related brain diseases. Clinical studies in Parkinson’s disease, stroke, and Alzheimer’s disease have generally evaluated acupuncture as an adjunct to medication or rehabilitation. Some studies have reported improvements in cognition, motor and nonmotor symptoms, and selected functional outcomes, although findings vary across diseases and outcome measures. A few imaging studies have also reported changes in cerebral glucose metabolism or neural activity, complementing symptom and functional assessments with preliminary observations of cerebral responses to acupuncture.

### 8.1. Parkinson’s Disease

PD is a chronic progressive neurodegenerative disorder characterized by motor symptoms such aesting tremor, rigidity, bradykinesia, and postural or gait disturbance. Non-motor symptoms, including depression, anxiety, sleep disturbance, constipation, dysphagia, cognitive impairment, and autonomic dysfunction, are also common [128]. PD mainly affects older adults. Epidemiological estimates suggest that at least 6 million people are affected worldwide [129], and modeling studies predict a marked increase in global burden by 2050 as populations age [130]. Current treatment relies largely on dopaminergic replacement, adjunctive rehabilitation, and device-based approaches. These strategies improve symptoms but do not reliably slow disease progression [131]. Levodopa remains central to pharmacotherapy, but long-term use is limited by complications such as dyskinesia. Treatment of non-motor symptoms is usually symptom-specific and often produces modest benefit [128,132]. Acupuncture has been investigated as an adjunct to standard treatment for its potential to improve motor and nonmotor symptoms.

Systematic reviews suggest an adjunctive role for acupuncture in PD. A systematic review of 42 studies (*n* = 2625) reported that acupuncture combined with conventional medication was associated with greater improvements in overall symptom scores, activities of daily living, and motor function than medication alone. Results varied by acupuncture modality and outcome, and total Unified Parkinson’s Disease Rating Scale (UPDRS) scores did not differ significantly between acupuncture and sham acupuncture. The included trials were generally of low methodological quality [133]. A later systematic review included 66 trials, of which 61 contributed to the meta-analysis. It reported improvements in motor function, activities of daily living, mood, treatment-related complications, and quality of life, as well as reduced Madopar dosage, with acupuncture-related therapies added to conventional medication [134]. A more recent network meta-analysis of 57 RCTs (*n* = 4262) compared several acupuncture-related interventions, including manual acupuncture, electroacupuncture, warm needling, and moxibustion. Selected regimens combined with conventional medication showed benefits for motor or depressive symptoms. The certainty of evidence was low to moderate for most comparisons [123].

In a multicenter RCT (*n* = 166), adding electroacupuncture to routine medication for 12 weeks produced a greater reduction in UPDRS total scores than medication alone, with a between-group difference still evident at week 24. Spontaneous bowel-movement frequency was also higher among participants with constipation [135]. A small randomized trial of 50 patients (48 analyzed) reported greater improvements in motor and sleep measures with electroacupuncture added to drug therapy than with drug therapy alone, whereas between-group differences were not evident for depression. Serum nitric oxide increased in both groups, with a smaller rise in the electroacupuncture group [136]. For dysphagia, a meta-analysis of 10 RCTs (*n* = 724) reported better swallowing outcomes with adjunctive acupuncture. A pooled analysis of three trials also found fewer pulmonary infections, although most studies in the review had a high or unclear risk of bias [137]. Small neuroimaging studies have also examined cerebral responses to adjunctive acupuncture in PD. FDG-PET and SPECT studies, each involving 10 patients, reported increases in regional cerebral glucose metabolism and blood flow, respectively, after five weeks of scalp acupuncture combined with medication [138,139]. A 12-week randomized fMRI study enrolling 41 patients with tremor reported changes in cerebellar, thalamic, and motor cortical activity. The true-acupuncture group also showed reductions in tremor and UPDRS scores from baseline [140]. These preliminary observations raise the possibility that acupuncture-related cerebral responses involve the metabolic support of neuronal activity, including the capacity to meet the changing energy demands of synaptic signaling in PD.

### 8.2. Stroke

Stroke results from a sudden interruption of cerebral blood supply, either ischemic or hemorrhagic, and causes neurological dysfunction [141]. Its economic burden is substantial: global stroke-related expenditure exceeds USD 890 billion, about 0.66% of global GDP, with rapid growth in low- and middle-income countries [142]. Acute ischemic stroke management focuses on reperfusion, whereas hemorrhagic stroke care aims to reduce rebleeding, hematoma expansion, and secondary injury. Reperfusion therapies are constrained by narrow time windows and unequal access, and hemorrhagic stroke often requires intensive monitoring and supportive care [143,144]. In older adults, disease burden remains high across prevention, acute care, and rehabilitation [145]. This creates a clinical rationale for accessible adjunctive strategies that can support recovery without replacing standard care.

Evidence syntheses report improvements in selected stroke outcomes, although heterogeneity remains a major limitation. A 2010 systematic review included 56 studies and pooled 38 trials, showing an overall effect favoring acupuncture over control interventions. Substantial heterogeneity, methodological limitations, and possible publication bias reduced confidence in the pooled estimate [146]. A later meta-analysis of 18 higher-quality RCTs (*n* = 1411) reported that electroacupuncture, compared with conventional Western medical treatment, improved self-care capacity, functional independence, motor recovery, and neurological deficit severity in acute ischemic stroke, with good tolerability [147]. For post-stroke spasticity, a systematic review of 22 RCTs (*n* = 1425) found that electroacupuncture added to routine care reduced upper- and lower-limb spasticity and improved activities of daily living, overall motor function, and lower-limb motor function within 180 days after stroke [148].

RCTs have reported improvements in neurological deficits, language function, activities of daily living, and selected motor outcomes with acupuncture added to post-stroke rehabilitation. In a multicenter prospective RCT of 250 patients with mild-to-moderate acute ischemic stroke, patients receiving 18 acupuncture sessions over 3 weeks in addition to standard rehabilitation showed greater improvements from baseline to week 7 in neurological deficit scores and lower-extremity function than those receiving rehabilitation alone. Among patients with swallowing or cognitive impairment at baseline, the respective outcomes also favored the acupuncture group [149]. In poststroke motor aphasia, a multicenter sham-controlled RCT of 252 patients receiving language training and conventional treatment showed greater reductions in language impairment and improvements in functional communication with manual acupuncture than with sham acupuncture at 6 weeks. These between-group differences remained at 6 months after onset. The manual acupuncture group also showed greater improvements in Stroke-Specific Quality of Life Scale and NIHSS scores than the sham acupuncture group [150]. An evaluator- and analyst-blinded multicenter RCT randomized 108 patients with convalescent-stage ischemic stroke to scalp acupuncture plus rehabilitation or rehabilitation alone. After 8 weeks, the combined intervention produced greater improvements in Fugl–Meyer Assessment, modified Barthel Index, and modified Rankin Scale scores. It also increased the fractional amplitude of low-frequency fluctuations (fALFF) in motor-related regions, including the cerebellum, precuneus, and precentral gyrus; baseline fALFF in the ipsilateral precentral gyrus showed the strongest correlation with Fugl–Meyer improvement [151]. Acupuncture has shown adjunctive benefits for selected functional outcomes after stroke, with preliminary imaging findings also indicating changes in motor-related brain activity. These observations provide a potential rationale for examining whether the mitochondrial processes implicated in experimental studies also contribute to recovery in patients receiving acupuncture.

### 8.3. Alzheimer’s Disease

AD causes progressive cognitive decline and increasing dependence in daily life. Globally, an estimated 57 million people were living with dementia in 2019. Cholinesterase inhibitors and memantine provide symptomatic treatment for AD [152]. Lecanemab and donanemab have slowed clinical decline in selected patients with early AD, although the benefits are modest and accompanied by a risk of amyloid-related imaging abnormalities [153,154].

A meta-analysis of 12 RCTs involving 893 patients found that acupuncture combined with donepezil improved cognitive scores and daily functioning compared with donepezil alone. The eight trials reporting Mini-Mental State Examination (MMSE) scores yielded a mean difference of 3.28 points (95% CI, 1.81–4.75). Substantial heterogeneity in the MMSE estimates (I^2^ = 86%) and the absence of participant blinding across the included trials limit confidence in the size of this additional benefit [155]. In an RCT of 87 patients with mild-to-moderate AD, 12 weeks of acupuncture was compared with donepezil. Scores on the Alzheimer’s Disease Assessment Scale–Cognitive Subscale (ADAS-Cog) favored acupuncture at the end of a subsequent 12-week follow-up, but daily functioning did not differ significantly between groups [156]. More direct evidence for an adjunctive cognitive benefit comes from a 66-patient trial comparing 24 weeks of EA with sham EA alongside standard pharmacological treatment. At week 24, the between-group difference in change in ADAS-Cog scores was −6.12 points (95% CI, −10.00 to −2.23), favoring EA. This advantage remained four weeks after treatment ended, whereas total activities-of-daily-living scores did not differ significantly at either assessment. The small sample and brief post-treatment follow-up leave the durability of the effect uncertain [157]. With functional benefits less consistent, current evidence is more encouraging for acupuncture as an adjunct to medication for cognitive symptom management in AD.

## 9. Limitations and Implications of Current Research on Acupuncture

Experimental studies have assessed mitochondrial structure and energy metabolism alongside neurological outcomes. Studies combining functional measurements with pharmacological or genetic interventions provide more direct support for some molecular targets in acupuncture-related protection. The associated mitochondrial processes, however, are still largely inferred from indirect measurements. Differences in disease models, brain regions, and treatment and sampling times complicate comparisons. How mitochondrial responses interact under different pathological conditions also remains unclear.

Where morphology has been assessed qualitatively, quantitative structural and membrane lipid analyses paired with functional measurements could clarify the extent and functional significance of mitochondrial preservation. Matched comparisons of ubiquitin-dependent and ubiquitin-independent mitophagy could distinguish their relative contributions to clearance. UPRmt, axonal mitochondrial transport, intercellular mitochondrial transfer, and mitochondrial–nuclear retrograde signaling also warrant further study [30,31]. Molecular profiling, quantitative assays, and high-resolution intravital imaging could characterize these responses and epigenetic regulation; targeted pathway inhibition or gene editing could then test whether the implicated processes contribute to functional recovery in disease-specific models.

Direct mitochondrial assessments in patients remain limited. Differences in acupuncture protocols and control conditions, together with incomplete reporting, also hinder comparisons. Preregistered, adequately controlled trials should pair prespecified clinical outcomes with repeated mitochondrial assessments selected for clinical feasibility and tissue specificity, alongside complementary measures of cerebral metabolism. Examining whether changes in bioenergetic capacity and stress tolerance precede or accompany clinical improvement could help determine whether acupuncture-specific mitochondrial responses influence susceptibility to neural injury and contribute to repair in patients.

## 10. Conclusions

Mitochondrial homeostasis shapes neural vulnerability and recovery in the aging brain through its roles in energy production, calcium buffering, organelle trafficking, and quality control. Across animal models relevant to chronic brain disease and acute brain injury, acupuncture has been associated with changes in mitochondrial ultrastructure, biogenesis, dynamics, mitophagy, redox balance, calcium handling, and apoptosis. These changes have been reported alongside improvements in neurological, behavioral, or injury-related outcomes, suggesting a possible link between the effects of acupuncture and mitochondrial homeostasis in these models. Reported improvements in selected symptoms and functional outcomes suggest an adjunctive role for acupuncture in Parkinson’s disease, stroke, and Alzheimer’s disease. The contribution of mitochondrial homeostasis to these clinical responses remains uncertain. Trials pairing prespecified clinical outcomes with feasible mitochondrial assessments, together with experimental tests of pathway necessity, could clarify whether acupuncture-associated mitochondrial responses influence the capacity of vulnerable neural tissue to meet energy demands, withstand stress, and support repair. Understanding these responses may help explain the biological basis of symptom relief and functional recovery, including why their extent and persistence vary across disease states and treatment protocols.

## Figures and Tables

**Figure 1:**
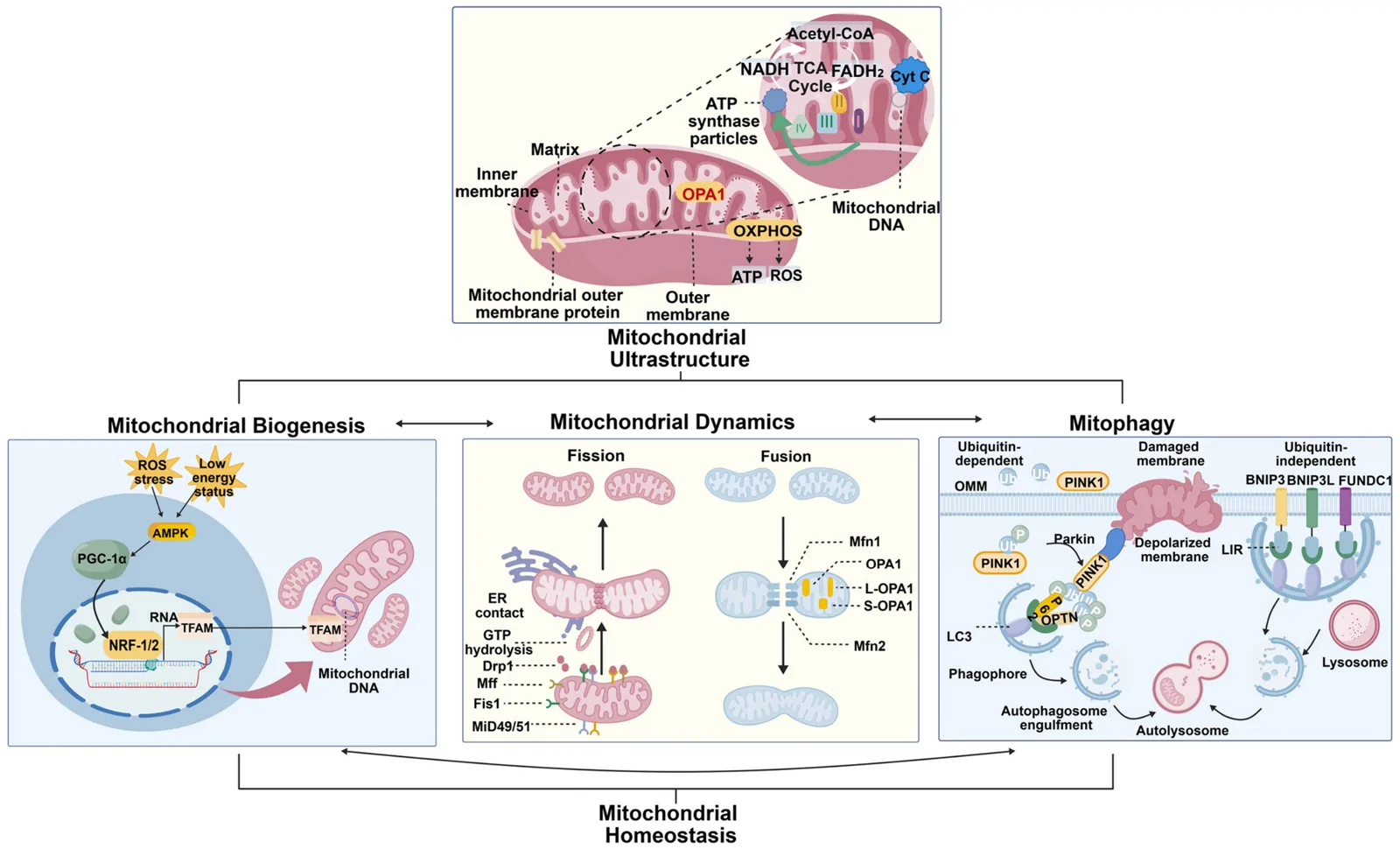
Mitochondrial homeostasis. Homeostasis is sustained by mitochondrial biogenesis, dynamics (fission/fusion), and mitophagy, which together maintain mitochondrial abundance and function. Dashed arrows denote annotations; unidirectional arrows represent steps in biological processes; bidirectional arrows suggest reciprocal influences between reactions. This figure was created with BioGDP.com [26].

**Figure 2:**
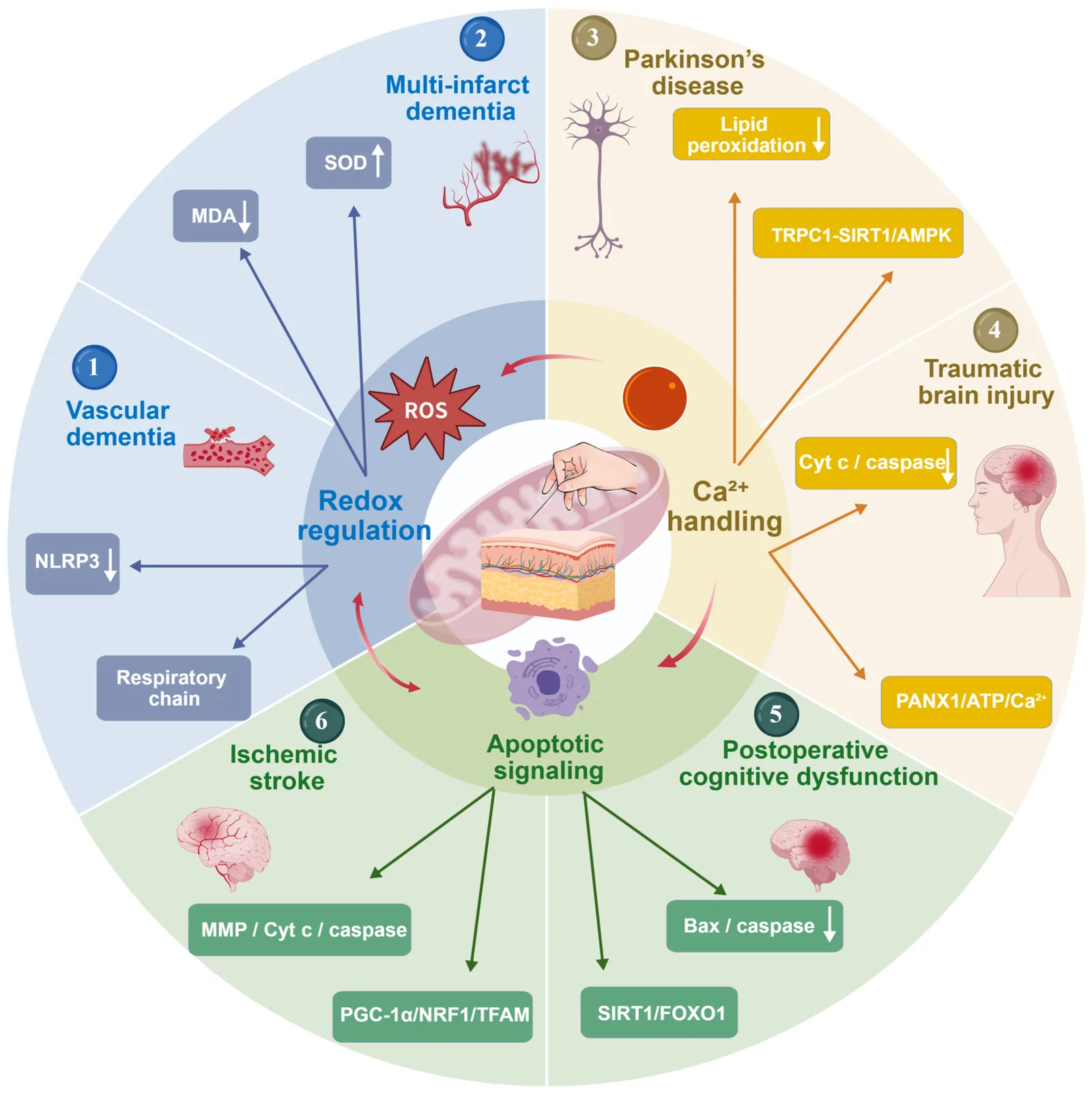
Acupuncture-related changes in mitochondrial redox regulation, Ca^2+^ handling, and apoptotic signaling reported in experimental models of brain disease. The diagram summarizes findings from acupuncture or electroacupuncture studies in experimental models of vascular dementia, multi-infarct dementia, Parkinson’s disease, traumatic brain injury, ischemic stroke, and postoperative cognitive dysfunction. Reported outcomes and signaling changes include respiratory-chain function, SOD/MDA balance, NLRP3 activation, lipid peroxidation, TRPC1-SIRT1/AMPK and PANX1/ATP/Ca^2+^ signaling, mitochondrial membrane potential (MMP, ΔΨm), cytochrome c/caspase-related apoptosis, PGC-1α/NRF1/TFAM signaling, and Bax/caspase expression. This figure was created with BioGDP.com [26].

**Figure 3:**
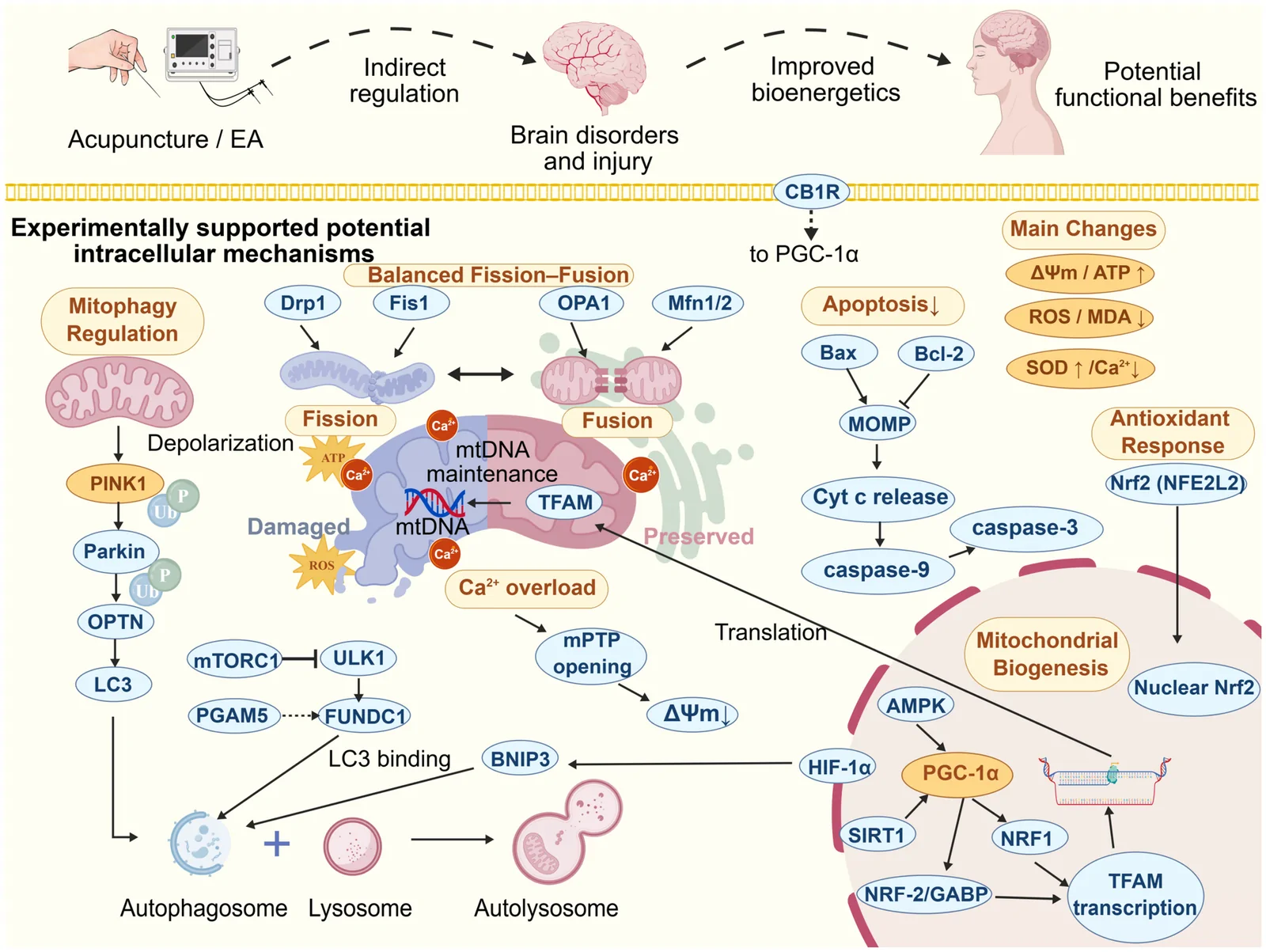
Potential mechanisms of acupuncture-associated mitochondrial protection in brain disorders and injury. Experimental studies associate acupuncture and electroacupuncture (EA) with preserved mitochondrial structure, improved bioenergetics, and reduced neuronal injury. The diagram summarizes representative mechanisms involving mitochondrial biogenesis, fission-fusion balance, mitophagy, antioxidant responses, Ca^2+^ regulation, and apoptosis. Mitophagy-related responses may vary across disease models and intervention schedules. The TFAM-directed arrow includes cytosolic translation and mitochondrial import. NRF-2/GABP is distinct from Nrf2 (NFE2L2). Solid arrows indicate regulatory relationships or process progression, blunt ends indicate inhibition, and dashed arrows indicate indirect or proposed links. The double-headed arrow denotes fission–fusion balance. ↑ and ↓ indicate representative changes relative to model controls; Ca^2+^↓ refers to reduced pathological Ca^2+^ overload. This figure was created with BioGDP.com [26].

**Table 1:** Acupuncture-associated changes in mitochondrial quality control in chronic brain disease and acute brain-injury models.

Mitochondrial Process	Reference	Disease Model	Modality	Intervention Protocol	Molecular Markers Assessed
Mitochondrial biogenesis (AMPK/PGC-1α axis)	[63]	MCAO (rat)	MA/GV16, GV20, ST36, LI11	20 min; once daily; 7 d	AMPK, PGC-1α, Nrf2, TFAM ↑
	[64]	OVX + CUMS (rat)	EA + neural stem cell-derived exosomes (NSCs-Exo)/GV20, BL23, KI3	Dense-Disperse wave; 15 min; once daily; 28 d	AMPK, p-AMPK, PGC-1α, NRF1, TFAM ↑
Mitochondrial dynamics	[65]	Ischemic stroke (mouse)	EA/ST36	Continuous wave; 15 Hz, 15 min; once daily; 3 d	Fis1, Drp1 ↓
	[52]	I/R (rat)	EA/GV20, GV14	2 Hz, 1.0 mA, 20 min; once daily; 7 d	Drp1, Fis1 ↓; Mfn1, Mfn2, OPA1 ↑
	[66]	ICH (rat)	MA/GV20, GB7 (through-needling)	Continuous wave rotation, 5 min; repeated every 5 min during retention; 30 min; once daily; 3 d	Fis1, mito-Drp1, Drp1-Ser637 ↑; Drp1-Ser616, OPA1 S/L ↓; Mfn1/2 ↑
PINK1/Parkin-mediated mitophagy	[67]	Embolic stroke (rat)	MA (*Xingnao Kaiqiao*)/GV26, PC6	manipulation, 1 min; needle retention, 30 min/session	PINK1, Parkin, LC3 ↑; COX4I1 ↓
	[68]	AD (mouse)	MA/GV20, KI1	manipulation every 5 min; 20 min; once daily; 40 d	SIRT3, PGC-1α, PINK1, LC3B ↑
	[69]	PD (mouse)	EA/GV26, PC6, SP6	Dense-Disperse wave; 5/20 Hz, 0.5 mA, 20 min; once daily; 30 d	Miro1, PINK1, Parkin ↓

Note: Scalp-acupuncture line mapping may vary across sources; ↑ indicates increased expression/level, and ↓ indicates decreased expression/level. Abbreviations are provided in Supplementary Tables S1 and S2.

## Data Availability

No new data were created or analyzed in this study. Data sharing is not applicable to this article.

## Supplementary Materials

The following supporting information can be downloaded at: https://www.mdpi.com/article/10.3390/brainsci16101016/s1, Table S1: General biomedical abbreviations; Table S2: Acupoint abbreviations.
