# Supplementary material for: Acupuncture in Aging-Related Brain Diseases: From Mitochondrial Homeostasis to Clinical Signals
Source: Brain Sci. 2026 Sep 24;16(10):1016. doi: 10.3390/brainsci16101016 (PMC13702579; doi:10.3390/brainsci16101016)
Supplement: Supplementary file 1 [file brainsci-16-01016-s001.zip › Supplementary Tables.pdf]

Supplementary Tables
List of Abbreviations

Supplementary Table S1. General biomedical abbreviations

Abbreviations are presented as abbreviation-full term and normalized for consistency.

Abbreviation	Full term
AD	Alzheimer's disease
AMPK	AMP-activated protein kinase
ATP	adenosine triphosphate
BAK	BCL-2 antagonist/killer
BAX	pro-apoptotic BCL-2-associated X protein
BCL-2	anti-apoptotic B-cell lymphoma/leukemia-2 protein
BNIP3	BCL2/adenovirus E1B 19 kDa-interacting protein 3
BNIP3L	BCL2/adenovirus E1B 19 kDa-interacting protein 3-like
Ca2+	calcium ion
CB-1R	cannabinoid receptor-1
CF	chronic fatigue
COX-IV	cytochrome c oxidase subunit IV
Cu/Zn-SOD	copper/zinc superoxide dismutase
CUMS	chronic unpredictable mild stress
Drp1	dynamin-related protein 1
E2	estradiol
EA	electroacupuncture
ER	endoplasmic reticulum
ETC	electron transport chain
EVs	extracellular vesicles
Fis1	mitochondrial fission 1 protein
FSH	follicle-stimulating hormone
FUNDC1	FUN14 domain-containing 1
GJCs	gap junction channels
H2O2	hydrogen peroxide
HIF-1α	hypoxia-inducible factor-1α
HT	hormone therapy
I/R	ischemia-reperfusion
ICH	intracerebral hemorrhage
IMM	inner mitochondrial membrane

Abbreviation	Full term
L-OPA1	long OPA1 isoform
LC3	microtubule-associated protein 1 light chain 3
MA	manual acupuncture
MCAO	middle cerebral artery occlusion
MDA	malondialdehyde
Mff	mitochondrial fission factor
Mfn1/2	mitofusins 1/2
MiD49	mitochondrial dynamics protein of 49 kDa
MiD51	mitochondrial dynamics protein of 51 kDa
Mn-SOD	manganese superoxide dismutase
mtDNA	mitochondrial DNA
NADH	nicotinamide adenine dinucleotide
NRF1	nuclear respiratory factor 1
NRF2	nuclear respiratory factor 2
OMM	outer mitochondrial membrane
OPA1	optic atrophy protein 1
OVX	ovariectomy
OXPHOS	oxidative phosphorylation
PCOS	polycystic ovary syndrome
PD	Parkinson's disease
PGC-1α	peroxisome proliferator-activated receptor gamma coactivator 1α
PINK1	PTEN-induced kinase 1
POI	premature ovarian insufficiency
ROS	reactive oxygen species
S-OPA1	short OPA1 isoform
SASP	senescence-associated secretory phenotype
SIRT-1	sirtuin-1
SOD	superoxide dismutase
TFAM	mitochondrial transcription factor A
TNTs	tunneling nanotubes
UPRmt	mitochondrial unfolded protein response
VMS	vasomotor symptoms

Supplementary Table S2. Acupoint abbreviations

Acupoint codes are listed with their corresponding acupoint names.

Code	Acupoint name
BL17	Geshu
BL23	Shenshu
BL54	Zhibian
CV3	Guanyuan
CV4	Zhongji
CV6	Qihai
CV12	Zhongwan
CV17	Danzhong
CV23	Lianquan
EX-CA1	Zigong
EX-HN1	Sishencong
EX-HN12	Jinjin
EX-HN13	Yuye
GB7	Qubin
GB20	Fengchi
GV14	Dazhui
GV15	Yamen
GV16	Fengfu
GV20	Baihui
GV23	Shangxing
GV26	Shuigou
KI1	Yongquan
KI3	Taixi
LI4	Hegu
LI11	Quchi
LR3	Taichong
PC6	Neiguan
SP6	Sanyinjiao
SP9	Yinlingquan
SP10	Xuehai
ST25	Tianshu

Code	Acupoint name
ST28	Shuidao
ST36	Zusanli
